# Inductive Displacement Sensor Operating in an LC Oscillator System Under High Pressure Conditions—Basic Design Principles

**DOI:** 10.3390/s25196078

**Published:** 2025-10-02

**Authors:** Janusz Nurkowski, Andrzej Nowakowski

**Affiliations:** Strata Mechanics Research Institute of the Polish Academy of Sciences, Reymonta 27, PL–30-059 Kraków, Poland; janusz.nurkowski@imgpan.pl

**Keywords:** inductive displacement sensor, optimization, sensor geometry, resonant system, LC oscillator, quality factor (*Q*-factor)

## Abstract

**Highlights:**

**What are the main findings?**

- Presentation of the basic design principles of an inductive displacement sensor for rock samples, enabling high-resolution measurements (≥10^−6^) in a high-pressure chamber under hydrostatic pressures of several hundred MPa and temperature variations of several tens of degrees Celsius.
- Discussion of the challenges and limitations related to sensor fabrication and the stability of the associated LC oscillator.

**What is the implication of the main findings?**

- The presented sensor facilitates rock deformation measurements under pressure and temperature conditions equivalent to those at depths of several kilometres, which is critical in the context of increasing resource-extraction depths and the development of underground fuel-storage facilities.
- The presented sensor offers a low-cost, simple-to-fabricate solution with favourable metrological properties, providing a viable alternative to conventional sensors (e.g., resistive strain gauges), which face limitations under such conditions.

**Abstract:**

The paper presents some design principles of an inductive displacement transducer for measuring the displacement of rock specimens under high hydrostatic pressure. It consists of a single-layer, coreless solenoid mounted directly onto the specimen and connected to an LC oscillator located outside the pressure chamber, in which it serves as the inductive component. The specimen’s deformation changes the coil’s length and inductance, thereby altering the oscillator’s resonant frequency. Paired with a reference coil, the system achieves strain resolution of ~100 nm at pressures exceeding 400 MPa. Sensor design challenges include both electrical parameters (inductance and resistance of the sensor, capacitance of the resonant circuit) and mechanical parameters (number and diameter of coil turns, their positional stability, wire diameter). The basic requirement is to achieve stable oscillations (i.e., a high *Q*-factor of the resonant circuit) while maintaining maximum sensor sensitivity. Miniaturization of the sensor and minimizing the tensile force at its mounting points on the specimen are also essential. Improvement of certain sensor parameters often leads to the degradation of others; therefore, the design requires a compromise depending on the specific measurement conditions. This article presents the mathematical interdependencies among key sensor parameters, facilitating optimized sensor design.

## 1. Introduction

The laboratory triaxial compression test is an experiment in rock mechanics that allows simulation, under laboratory conditions, of the stress environment in rock masses at various depths (even several kilometres deep). Measuring the deformation of the specimen during such a test is essential for determining the deformational properties of the tested material and therefore must be carried out directly on the specimen, inside the high-pressure chamber.

This publication refers to a transducer designed to measure the deformation of a rock sample during two different experiments:

1. The conventional triaxial compression test, in which a geometrically defined (typically cylindrical) rock sample is first subjected to hydrostatic pressure corresponding to the geostatic stress at a given depth. Subsequently, an axial force is applied to the sample, at least until failure (i.e., reaching the critical stress and strain) or, if necessary, even beyond that point. The principles of performing this test—known as the individual test—were formulated by a team led by Professor Kálmán Kovári from the Zürich University of Technology and have since been widely accepted in rock mechanics (see [1]).
2. The compressibility test, which involves measuring the volumetric changes in a rock sample under the influence of hydrostatic pressure alone (without applying axial force). Detailed information on the execution of this experiment can be found in [2].

A key component of the experimental apparatus used in triaxial compression tests is the so-called triaxial cell (also referred to as a high-pressure cell), in which the tested sample is placed. This chamber is filled with a fluid (usually liquid) that applies hydrostatic pressure to the sample, and it also features a piston that enables the application of axial loading.

It is important to note that there is no such thing as a standardized triaxial cell. These devices are typically custom-built for specific research teams and may have significantly different technical parameters. The analyses and recommendations presented in this article are based on experimental studies conducted using the GTA-10 triaxial chamber, located at the Strata Mechanics Research Institute of the Polish Academy of Sciences in Kraków (Poland). This chamber allows for testing cylindrical rock samples under conventional triaxial stress conditions at a confining pressure of up to 400 MPa. The internal diameter of the triaxial chamber in this device is 50.0 mm. Kerosene, which is a physiochemically inert, non-corrosive and non-conductive liquid, was used as the fluid used to induce hydrostatic pressure. A detailed description of the GTA-10 apparatus can be found in [3,4].

Similarly, there are no standard, universal sensors for measuring the displacement of a sample placed inside the cell. Such sensors must be selected (or newly designed) taking into account the following parameters: the range of pressures and temperatures applied within the cell, the expected range of measured deformations, the anticipated movement range of the press piston, the available space in the chamber after placing the sample, and the remaining space after the deformation process is completed. These parameters are generally closely correlated.

For a deformation sensor used in a high-pressure cell it is necessary to fulfil following requirements:

(i) Resistance to high pressure.
(ii) Acceptably small impact of temperature changes on sensor readings.
(iii) A large measurement range (on the order of tens of millimetres) combined with high resolution and accuracy.
(iv) Compact dimensions (not exceeding a few millimetres in size).

While points (i) and (ii) are self-explanatory, points (iii) and (iv) require further discussion.

In a synthetic analysis of the behaviour of rock samples under conventional triaxial stress conditions using the GTA-10 device [5] demonstrated that the critical axial strain of a sample could reach 30%, with maximum values up to 50%. At the same time, lateral strain could be equal to axial strain.

Such strain values were observed during conventional triaxial compression tests on samples with a height of 45.0 mm and a diameter of 22.5 mm. This meant that the measurement range of the axial displacement sensor had to be at least 22.5 mm, while the circumferential displacement sensor required a range of at least 35.4 mm.

Conversely, during the compressibility test, the volume change in the sample rarely exceeded 1%. For an isotropic and homogeneous material, this corresponded to a linear strain of approximately 0.33%, necessitating a strain measurement resolution of at least 10^−5^, despite temperature fluctuations of several or even more than a dozen degrees Celsius caused by pressure variations. Considering all these factors, it is justified to expect that the designed dis-placement sensor must meet requirement (iii).

Taking into account the so-called scale effect and the need to reduce the influence of local sample heterogeneities on the test results, it is desirable that the tested sample be as large as possible. This issue has been thoroughly discussed in the works of [6,7,8,9]. On the other hand, the size of the pressure cell—and consequently, the size of the sample placed inside—is limited by the strength of the steel from which the cell is made. As a result, to make optimal use of the cell, the installed deformation sensors should occupy as little space as possible, which leads to the fulfilment of requirement (iv).

In the Rock Deformation Laboratory of the Strata Mechanics Research Institute of the Polish Academy of Sciences, during the tests conducted on the GTA-10 device the dimensional changes in the sample are primarily measured, subjected to either the conventional tri-axial compression test or the compressibility test. For these tests, an inductive displacement-to-frequency transducer developed in the laboratory is used as a deformation sensor. This transducer operates in an LC oscillator circuit (Colpitts type) and meets all four requirements (i)–(iv) outlined earlier.

The sensor consists of a single-layer, coreless inductive coil, made of thin, flexible wire and attached directly to the sample. It serves as the inductance component of an LC resonant circuit oscillator, which is located outside the pressure cell. As the sample deforms, the sensor changes in length, altering its inductance, which in turn changes the oscillation frequency. These frequency changes are converted into dimensional variations in the sample. The sensor offers a large measurement range and high resolution, has a compact design, is resistant to high hydrostatic pressure, and is easy to manufacture.

The first reports on the sensor’s operation and the results obtained using it were published by [10]. Both the sensor and its measurement methodology are continuously being developed, with various metrological aspects of their application discussed, among others, in the following works: [11,12].

**Figure 1:**
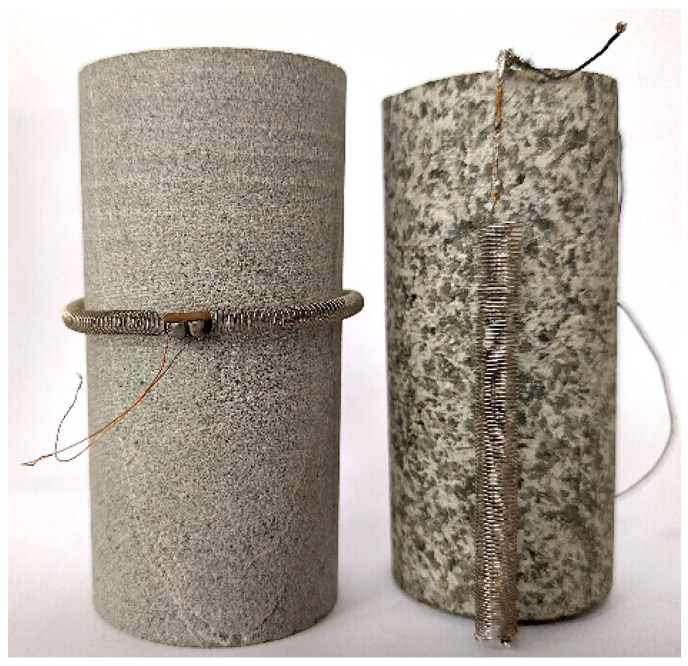
Method of mounting the inductive deformation sensor on the specimen: left—in a conventional triaxial compression test; right—in a compressibility test.

Figure 1 presents two versions of the sensor. On the left, the sensor is shown in the form of a torus encircling the sample at mid-height, used to measure the circumferential deformation of the sample in the conventional triaxial compression test. On the right, the sensor is shaped like a simple spring soldered to metal clamps, which are attached to the tested sample. In this configuration, the sensor is used during the compressibility test.

**Figure 2:**
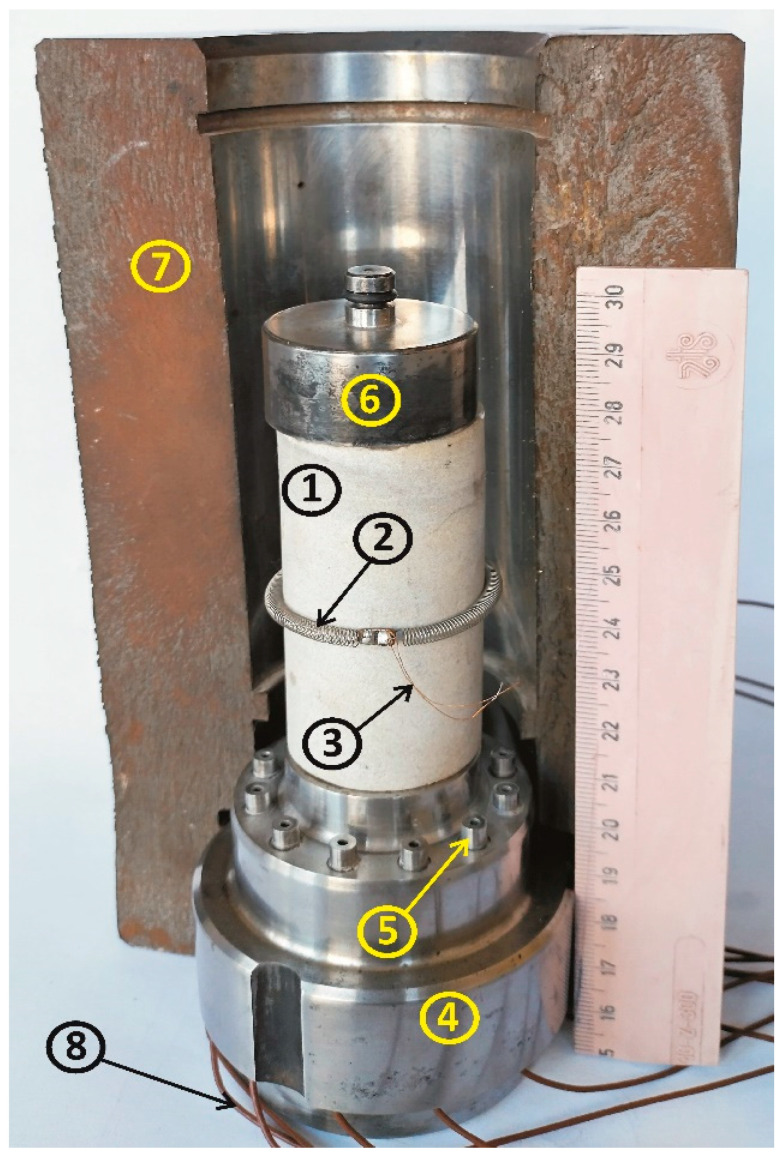
Rock specimen with a mounted inductive circumferential deformation sensor inside the high-pressure cell (cross-sectional view of the chamber). Figure legend: 1—rock specimen; 2—circumferential deformation sensor (toroid); 3—wires connecting the sensor to the feedthroughs in the high-pressure cell plug; 4—high-pressure cell plug; 5—electrical feedthroughs; 6—anvil; 7—high-pressure cell (cross-section); 8—wires connecting the plug feedthroughs to the LC generator.

Figure 2 shows the toroidal sensor in the configuration in which it operates inside the high-pressure cell. It should be noted that the sample (1) shown in the figure has a diameter of 32.0 mm, while the high-pressure cell (7) has a diameter of 50.0 mm. It means that the gap between the sample and the cell is 9.0 mm. Within this space, both the sensor and the deforming sample must fit. This highlights the importance of the sensor’s small dimensions (see condition (iv) above). In practice, samples with a 32.0 mm diameter have been used very rarely, while the standard samples have had a 22.0 mm diameter, which provided 14.0 mm of space for the sensor and the sample’s circumferential deformation.

The aim of this article is to develop a description—based on previous experience—of the criteria and method for selecting both the electrical and mechanical optimal parameters of the displacement sensor used for measurements in the GTA-10 device (produced by Institute of High-Pressure Physics of the Polish Academy of Sciences UNIPRESS, Warsaw, Poland). It has been demonstrated that these parameters are interrelated, necessitating a compromise between them.

In particular this applies to parasitic capacitances, especially of the electrical feedthroughs in the pressure chamber, and to the parasitic inductances of the sensor-oscillator connections. These two factors, whose values depend on pressure and temperature, destabilize oscillations and thus cause measurement errors.

The first design principle adopted for the sensor was to maximize the quality factor of the resonant circuit in which the sensor operates. This quality factor ensures the necessary stability and amplitude of oscillations. It is directly proportional to the sensor’s inductance and inversely proportional to the capacitance of the resonant circuit. Therefore, in order to reduce the influence of parasitic inductances by increasing the sensor’s inductance without decreasing the quality factor of the resonant circuit, the capacitance of the circuit must be decreased, which in turn amplifies the destabilizing influence of parasitic capacitances.

The second design principle was to select the sensor’s geometry and tension force in such a way as to maintain a stable position of the coil turns relative to each other during the experiment, and to prevent unstable operation due to material creep in the clamps or in the connections between the clamps and the sensor.

As a result of this targeted design and manufacturing process, a displacement sensor with the following properties was obtained:

(a) Measurement resolution on the order of 100 nm; this result was achieved for rock samples under hydrostatic pressure conditions of several hundred MPa, with temperature variations in several tens of degrees Celsius.
(b) The relative measurement error depends on the ratio of sensor sensitivity to the sample length. If the measurement uncertainty for a 10 mm-long sensor is 1000 nm, and the sensor is mounted (using a thin rod) on a 100 mm-long sample, then the relative uncertainty will be 10^−5^. For a longer gauge length, the relative uncertainty will be correspondingly smaller.
(c) The sensor is resistant to hydrostatic pressure; it was tested under the maximum hydrostatic pressure available in the laboratory—up to 1500 MPa—and it is presumed that it will operate correctly under even higher pressures. The sensor is routinely used under hydrostatic pressures up to 400 MPa.
(d) The sensor is resistant to elevated temperatures; it operated correctly at temperatures up to 250 °C. It was not tested at temperatures above 250 °C due to the use of lead-tin solder in its connections. Using other types of connections, such as welded joints, would significantly increase the allowable temperature range.
(e) To ensure the required measurement accuracy under extreme conditions, a comparative measurement method involving a reference sensor was developed [11]. The reference sensor is identical to the measuring sensor and is mounted on a steel support with known compressibility and thermal expansion. Both sensors are placed inside the pressure chamber, so temperature and pressure affect the sensors and electrical feedthroughs in the chamber wall almost identically. So, the readings from the reference sensor allow for correction of the measuring sensor’s output due to pressure and temperature changes in the chamber. Moreover, the measuring and reference sensors are alternately connected to the same oscillator, thereby virtually eliminating the influence of temperature fluctuations and supply voltage variations on the oscillator. A detailed description of such measurements can be found in [11].

The sensor developed and tested by the authors can successfully replace strain gauges, whose use is problematic under the conditions described above.

The novelty of the displacement-to-frequency transducer developed and experimentally verified by the authors lies in its accessibility: the device can be fabricated independently by any user at minimal cost, while providing the performance of a high-grade measuring instrument.

The sensing element (coil) enables, as demonstrated above in (a)–(e), displacement measurements of about 20 mm with an accuracy of 10^−5^, when subjected to temperature changes on the order of tens of degrees Celsius and hydrostatic pressures up to 1.5 GPa. It should be emphasized that the sensor was tested under hydrostatic pressures up to 1.5 GPa (which resulted from the limitations of the available equipment), but in the authors’ opinion—due to the simplicity of its design and its resistance to damage—it is difficult to theoretically predict the upper limit of its applicability. The sensor is characterized by mechanical simplicity, compact geometry, and ease of dimensional adaptation to specific application requirements.

From the electrical standpoint, the Colpitts oscillator represents a well-established electronic circuit, widely recognized for its robustness and ease of implementation. Its additional advantage is the inherently digital nature of the output signal: the measurable parameter is the frequency, which can be determined both precisely and conveniently, thereby eliminating the necessity of employing an A/D converter.

In their article, the authors endeavoured to articulate the fundamental design principles that a prospective user of the sensor/coil should follow when attempting to develop such a device for their own applications.

## 2. Review of Some Available Measurement Methods and Devices

Measuring the deformation of a rock sample under high-pressure conditions of several hundred MPa, accompanied by temperature variations in several dozen degrees, is a non-trivial task. Various solutions to this problem can be found both in scientific literature and in commercial product offerings.

Historically, the first discussions on this topic appeared in the works of [13,14] suggesting the use of resistance strain gauges which are glued directly onto the surface of the tested sample. The strain of the sample results in resistance changes in the gauge. The use of resistance strain gauges under high-pressure conditions has been discussed, among others, by [15,16,17,18,19]. Some issues related to this application were analyzed also by [20,21,22,23]. The disadvantage of such a method is the small range of deformation measured (a few per cent at most). If the sensor is glued to a rock sample, there is a risk that the pressure will dent the resistive path in a cavern or fracture in the rock, making it impossible, or worse, falsifying the measurement.

The second group of resistive deformation sensors used under high-pressure conditions includes the so-called Local Deformation Transducer (LDT). In these devices, a resistance strain gauge is attached to an elastic steel strip, which serves as a displacement reducer. This makes it possible to measure larger deformations, but measuring small ones can be difficult. Examples of such solutions were presented by [24,25,26].

Another notable group of sensors includes solenoidal sensors, which rely on changes in the inductance of a coil depending on the displacement of a ferromagnetic core inside it. A widely used device in this category is the LVDT (Linear Variable Differential Transformer). It consists of three individual coils wound concentrically around a hollow, non-magnetic, insulated tube. One coil serves as the primary winding, supplied with a sinusoidal signal of constant amplitude and frequency (typically between 1 kHz and 10 kHz), while the other two coils act as identical secondary windings. The magnetic flux generated by the primary winding is transferred to the secondary windings. These secondary windings are connected in series but in opposite phases, meaning that the output voltage is the difference between the induced voltages in each. If the core is in the central position the system is balanced, no voltage appears at the output of the secondary windings. However, when the core shifts, the system loses balance, resulting in an output voltage proportional to the displacement. Examples of commercial devices of this type include the S100.0 sensor manufactured by Solartron Metrology [27] and the CD 375 series sensors produced by Macro Sensors [28].

The use of LVDT sensors for deformation measurements under high-pressure conditions encounters numerous challenges and has an extensive bibliography. This topic has been discussed by authors such as [25,29,30,31,32].

Deformation measurement methods based on inductive-frequency transducers (which are almost identical to LVDT sensors) continue to evolve. There are two fundamental technical solutions used in the design of such sensors:

(1) Fixed core, moving coil—this version was discussed in detail by the authors in the separate article.
(2) Moving core, fixed coil—in this case, two coils with cores are connected to two oscillators, and the output signal consists of two frequencies. Each core moves inside its respective coil in such a way that when the inductance of the first coil decreases, the inductance of the second coil increases. A frequency difference is calculated in the processor, enabling linearization of the displacement-frequency characteristic and temperature compensation in the measurement (for details, see [33,34,35]).

An improved version of the type (2) converter was published by [36,37] under the name Differential Inductive Frequency Output Displacement (DIFOD). DIFOD achieved similar sensitivity and stability to the spring sensor, which is the subject of this article. How-ever, the spring sensor has the advantage that its coil length changes without friction, whereas in DIFOD (as in LVDT sensors), eliminating friction between the core and the coil frame-work remains difficult.

It is also worth mentioning that, in the case of displacement-to-frequency transducers, the traditional discrete oscillator can be replaced with integrated circuits from the LDC1000 series by Texas Instruments. Examples of such implementations have been demonstrated by [36,37].

In recent years, no fundamental breakthrough has been achieved in the measurement of rock sample deformation in high-pressure cells. Reference [38] presented an inductive sensor designed for measuring circumferential strain of a sample in such a cell; however, this is essentially a conventional inductive LDT, whose dimensions preclude its use in smaller-diameter cells such as the GTA-10. A particularly interesting approach, especially for measuring very small strains, was proposed by [39], who employed optical fibres affixed to the sample surface as displacement sensors. This method, however, requires highly sophisticated auxiliary equipment (a laser source for the optical fibres and an interferometer for signal analysis). Reference [40] modified in their work the method of [41] for calculating transverse strains of a sample from its volumetric change, which in turn represents an update of the concept originally introduced by [42]. Finally, the two most recent papers on triaxial compression tests are those of [43,44]. Both provide detailed descriptions of the design and operation of highly specialized triaxial cells but unfortunately omit information on the strain measurement techniques employed (apart from a brief note stating that LVDTs were used).

As for the market offer, the situation does not appear to be any better. Indeed, there are LVDTs with metrological parameters comparable to the sensor developed by the authors, but their dimensions make it impossible to use them in chambers of the GTA-10 size. A good example in this regard is [45].

## 3. Operating Principle of the Inductive Displacement Sensor, Its Design, and Design Limitations

**Figure 3:**
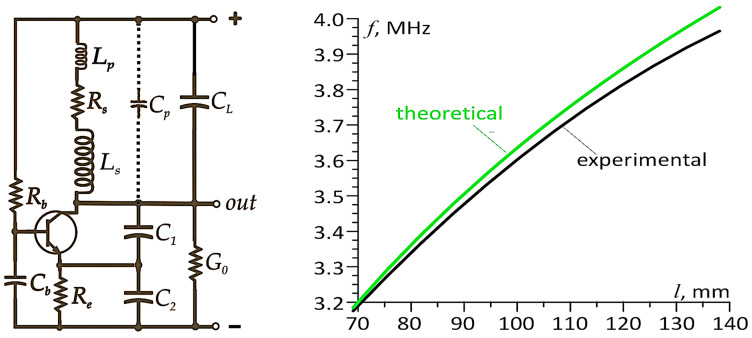
Sensor *L_s_* connected to the Colpitts oscillator (**left**), and an example of its characteristic response (**right**). Discrepancies between the theoretical curve (obtained from mathematical equations) and the experimental results arise from inaccuracies in estimating the parasitic capacitance *C_p_* and inductance *L_p_* of the connections; *C_b_*, *R_b_*, *R_s_*, *R_e_*—components necessary for the operation of the LC oscillator that do not directly affect the resonant circuit and are not taken into account in these considerations.

Figure 3 presents a schematic diagram of a Colpitts oscillator circuit with a split capacitance *C*_1_, *C*_2_ in the resonant circuit (see, e.g., [46,47,48]). The inductive component of the resonant circuit is the sensor under discussion. Additionally, the figure illustrates a typical relationship between the sensor length *l_s_* and the oscillation frequency of this circuit (the so-called transducer characteristic). The schematic also depicts parasitic inductances *L_p_* and capacitances *C_p_* of the sensor’s connections to the oscillator, as well as the sensor resistance *R_s_*. The capacitor *C_L_* can in some cases limit the influence of temperature on the oscillation frequency; *G*_0_ represents the load conductance of the oscillator.

The oscillation frequency *f* of such a resonant circuit is given by the following equation:

(1) $$f=\frac{1}{2\pi \sqrt{(L_{s}+L_{p})\cdot C}}=\;\frac{1}{2\pi \sqrt{(\frac{\mu \;z^{2}A}{l_{s}}+L_{p})\cdot C}}\;$$

where

*L_s_*, *L_p_*—inductances of the sensor itself and the parasitic inductance of the connections to the oscillator, respectively.

*C*—total capacitance of the resonant circuit.

*l_s_*—length of the displacement sensor.

*μ*, *z*, *A*—magnetic permeability, number of coil/sensor’s turns, and cross-sectional area of the coil/sensor, (for an air-core coil, the permeability can be assumed as *μ* = *μ*_0_).

It should be emphasized at this point that for certain values of *R*, *L*, and *C* in the resonance circuit, a significant reduction in the influence of the sensor’s temperature on the oscillation frequency (thermal compensation) is possible, as demonstrated by authors in [12]. The influence of temperature and pressure on the sensor readings can also be reduced by using the so-called reference measurement, which was described in detail by [11].

**Figure 4:**
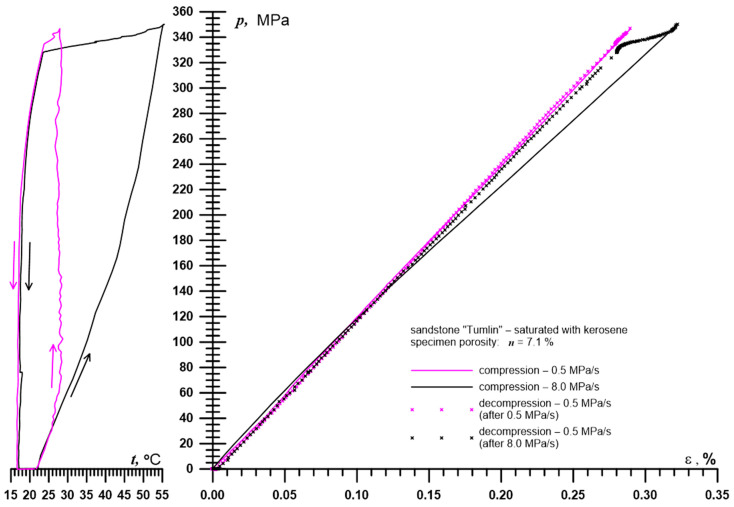
Example of a compressibility test result (for a detailed description of this type of test, see [49]);^.^ *P*—hydrostatic pressure in the high-pressure cell, *ε*—linear deformation of the sample, *t*—temperature in the high-pressure cell. Volume change in the sample *e* = 3*ε*. Arrows’ description: **↑↑**—compression, **↓↓**—decompression.

An example of strain measurement results using an inductive sensor operating in a system with a reference sensor is presented in Figure 4. Since the sensor is placed in a pressure chamber and the rest of the resonant circuit is outside it, it is not possible to use capacitors with an appropriate thermal coefficient of capacitance to compensate for the effect of temperature on the resistance and inductance (via thermal expansion of the wire) of the sensor and thus on the oscillation frequency.

The measurements were performed using a sensor characterized by the following parameters: length *l_s_* = 20.0 mm, diameter *D* = 4.0  mm, wire diameter *d* = 0.2 mm, and number of turns *z* = 60. The inductance of the sensor was *L_s_* ≈ 3.0  μH. The total capacitance of the resonant circuit was *C* = 1.1  nF, while the cumulative parasitic capacitance of the electrical pressure feedthroughs was approximately *C_p_* = 120  pF.

Based on this, the conditions in which the presented sensor must operate and its required measurement range can be determined. In this experiment, the hydrostatic pressure *p* applied to the tested sandstone sample varied from 0 to 350 MPa, with the sample being loaded at two different rates: 0.5 MPa/s and 8.0 MPa/s, while in both cases, unloading occurred at the same rate of 0.5 MPa/s. The temperature *t* measured during the experiment in the high-pressure cell varied accordingly from 16.6 °C to 28.7 °C and from 17.0 °C to 55.5 °C. The calculated maximum volumetric strain of the sample *e* was 0.97%. In reality, the sensor measured a linear strain of approximately 0.32%, which—assuming the homogeneity and isotropy of the tested rock—resulted in volumetric strain three times greater. The measurement base (sample length) was 50.0 mm, meaning that it was necessary to measure a length change of approximately 0.165 mm with resolution of at least 1000 nm. Such measurements were also made on granite samples, during which the measured deformations were almost ten times smaller.

The sensor, in the form of an inductive coil, which from a mechanical point of view is a helical tension spring, should meet the following design assumptions:

- The coil’s diameter should be as small as possible (a few millimetres) to minimize space usage in the pressure chamber.
- The coil should be as short as possible so that changes in the sample length result in significant relative changes in the sensor length, *ergo* significant relative changes in oscillation frequency.
- The coil must be resistant to mechanical shocks to allow repeated use without the risk of permanent deformation during measurements and mounting on the tested material.
- The coil mounted on the specimen must be pre-tensioned to prevent contact or friction between adjacent turns.
- The coil is single-layered.
- The coil is coreless; this avoids the influence of pressure and temperature variations on the magnetic permeability of the core and its unstable positioning relative to the coil.
- It is desirable to achieve the highest possible coil inductance relative to the inductance of its connections to the generator so that parasitic inductances of the connections do not destabilize the oscillations of the resonance circuit.
- The spacing between the coil turns should be uniform so that even under slight stretching, the turns do not touch, preventing inductance instability due to friction and electrical short circuits.
- The coil should have low resistance to ensure high quality factor (*Q*-factor) of the resonance circuit, which determines the stability and required amplitude of oscillations.

Additionally, it is desirable to achieve the highest possible capacitance of the resonance circuit relative to the capacitance of the coil connections to the oscillator, to prevent parasitic capacitances of the connections from destabilizing the circuit’s oscillations. In compressibility measurements, this particularly applies to the electrical capacitance of pressure feedthroughs in the cell plug, whose value depends on pressure and temperature changes.

The above requirements are often mutually contradictory. For example:

- A short sensor has high sensitivity but low inductance, which results in a greater destabilizing effect of the parasitic inductances of its connections to the resonant circuit.
- Attempting to increase the sensor’s inductance by enlarging the diameter of its turns risks their unstable positioning in the plane perpendicular to its axis, potentially causing electrical short circuits.
- Reducing the destabilizing effect of parasitic capacitances by increasing the capacitance of the resonance circuit decreases its *Q*-factor, which may lead to too low an oscillation amplitude or necessitate an increase in the sensor’s inductance, which is not always possible.
- Increasing the sensor wire’s diameter to reduce its resistance leads to increase the sensor’s tension force and decreases the number of (assuming the same sensor length) turns, which in turn reduces its inductance and increases the sensor’s tension force.
- A sensor resistant to mechanical shocks must be made of steel wire, which results in high resistance—especially in the case of spring wire—and also increases the tensile force exerted by the spring on the sensor mounting.

The following sections of this article demonstrate the consequences that changes in certain fundamental electrical and mechanical parameters of the sensor and the resonance circuit may influence on the entire measurement system, as well as how these parameters can be optimized.

## 4. *Q*-Factor and Sensitivity of the Sensor vs. Geometric Parameters of the Sensor

A necessary condition for oscillations to occur in the resonance circuit is that its *Q*-factor (*Q*) must be greater than 0.5 (Equation (2)):

(2) $$Q=\frac{1}{R}\sqrt{\frac{L}{C}}>0.5$$

In this formula, *R*, *L*, and *C* represent, respectively, the total resistance, inductance, and capacitance of the resonance circuit, including passive and active components of the electronic system as well as connections (such as electrical feedthroughs in the plug of the pressure cell) that influence the circuit. In the following considerations, it is assumed that the *Q*-factor of the resonant circuit is determined only by the coil resistance, which appears to be an acceptable simplification.

The condition *Q* = 0.5 is a limiting theoretical criterion; in practice, the higher the *Q*-factor of the circuit, the greater the stability and amplitude of oscillations. This is because a higher *Q*-factor results in a steeper phase characteristic near resonance. Therefore, according to Equation (2), it is desirable for the coil’s inductance to be as high as possible, while its resistance and the capacitance of the resonance circuit (capacitors *C*_1_ and *C*_2_) should be as low as possible. On the other hand, this capacitance should be big enough that the unstable capacitances of the connections, feedthroughs, and other components of the oscillator allow measurement of strain with sufficiently small uncertainty. During tests in the GTA-10 chamber, the total value of these unstable capacitances was approximately 10 pF, meaning that the resulting capacitance of the divider capacitors *C*_1_, *C*_2_ (Figure 3) should be at least 1000 pF. Under these conditions, it is possible to measure with an uncertainty of approximately 10^−6^ m, but the influence of temperature and pressure variations in the chamber on the sensor increase the uncertainty to 10^−5^ m.

Below is a mathematical analysis of how changes in the geometric parameters of the sensor affect its electrical characteristics.

### 4.1. Change in Sensor Coil Length

Let us consider the case of *n* series-connected identical coils. In such a system, the total inductance and resistance increase *n* times, and the quality factor *Q_n_* is given by the equation:

(3) $$Q_{n}\;=\;\frac{1}{nR}\sqrt{\frac{nL}{C}}$$

Now, calculating the ratio of *Q_n_* to *Q*, we obtain:

(4) $$\frac{Q_{n}}{Q}\;=\;\frac{\frac{1}{nR}\sqrt{\frac{nL}{C}}}{\frac{1}{R}\sqrt{\frac{L}{C}}}\;=\;\frac{\sqrt{n}}{n}=\frac{1}{\sqrt{n}}$$

This means that increasing the coil length n times results in a decrease in the quality factor of the resonant circuit by the square root of *n*.

Surprisingly, this scenario can still be considered advantageous, because the increase in sensor inductance comes at the cost of only a relatively small decrease in the quality factor. However, it is important to keep in mind, that: firstly—the sensor length is limited by the sample length, and secondly—increasing the coil/sensor length leads to a decrease in the sensitivity *s* of the entire transducer.

### 4.2. Sensitivity of the Sensor

The fundamental parameter indicating the quality of the sensor is its relative sensitivity *s*. It is defined as the relative change in the oscillation frequency *f* of the resonant circuit due to a change in the sensor length *l_s_*. Based on Equation (1), we obtain:

(5) $$\begin{array}{l} \;\;s=\frac{1}{f}\cdot \frac{df}{dl_{s}}=2\pi \sqrt{(\frac{\mu \;z^{2}A}{l_{s}}+L_{p})\cdot C}\;\cdot \;\;\frac{d\left(\frac{1}{2\pi \sqrt{(\frac{\mu \;z^{2}A}{l_{s}}+L_{p})\cdot C}}\right)}{dl_{s}}=\\ \;=\frac{k}{l_{s}^{2}}{\left(\frac{k}{l_{s}}+L_{P}\right)}^{-\left(\frac{3}{2}\right)}{\left(4\pi \sqrt{C}\right)}^{-1}\cdot 2\pi \sqrt{\left(\frac{k}{l_{s}}+L_{p}\right)C}=\\ \;=\frac{L_{S}}{2l_{s}\;(L_{s}+L_{p\;}\;)}=\frac{1}{2l_{s}\left(1+\frac{L_{p}}{L_{s}}\right)}\;,\;\;\mathrm{for}\;\;k=\mu \;z^{2}A \end{array}$$

It is easy to observe that the shorter the sensor, the higher its relative sensitivity, since the strain of the sensor (i.e., the change in length relative to its initial length) is greater. However, the sensor cannot be too short, because its inductance would then be too low, which reduces the quality factor of the resonant circuit (see (2)), leading to poorer oscillation stability. At the same time, the destabilizing influence of variable inductance in the connections increases. The inductance of the connections also reduces the sensor’s sensitivity (see (5), factor *L_p_*/*L_s_*). On the other hand, an attempt to increase the quality factor of the resonant circuit—after reducing the sensor’s length—by decreasing the circuit’s capacitance leads to oscillation instability due to the influence of the variable inductance in the connections.

From the above, it follows that the sensor’s sensitivity is limited by its resistance, as well as by the parasitic inductances and capacitances of the connections and the variability of their values.

### 4.3. Increasing the Diameter of Sensor Coil Turns

A potential way to increase the inductance of a coil is by increasing the diameter of its turns. In this case, the inductance increases while the coil length remains unchanged. As a result, the transducer’s sensitivity stays the same, while the capacitance of the resonant circuit can be increased, reducing the destabilizing effect of parasitic capacitances.

If a coil of a length *l_s_* is made from wire with thickness *d*, has *z* turns of the diameter *D*, its inductance is given by:

(6) $$L\;=\;\frac{\mu \;z\;A}{l_{s}}\;=\;\frac{\mu \;z\;\pi \;D^{2}}{4l_{s}}\;\mathrm{and}\;A\;=\;\frac{\pi \;D^{2}}{4}$$

Now, if the turn diameter is increased *n* times, the new inductance *L_n_* will be:

(7) $$L_{n}\;=\;\;\frac{\mu_{0}\;\mu_{r}z\;\pi \;{\left(nD\right)}^{2}}{4l_{s}}$$

Dividing Equation (7) by (6), we obtain:

(8) $$\frac{L_{n}}{L}\;=\;\frac{{\left(nD\right)}^{2}}{D^{2}}\;=\;n^{2}$$

This shows that increasing the turn diameter n times results in an *n*^2^-fold increase in the coil’s inductance. Meanwhile, the resistance *R_n_* of the coil with enlarged turns changes relative to the initial resistance *R* as:

(9) $$\frac{R_{n}}{R}\;\to \;\frac{n\;D}{D}\;=\;n$$

meaning that the resistance increases only *n* times (wire length increases *n*-times). Consequently, the relationship between the quality factor of the resonant circuit for the sensor with enlarged turns’ diameter *Q*(*n*∙*D*) and the quality factor for the primary sensor *Q*(*D*) is:

(10) $$\frac{Q(n\cdot D)}{Q(D)}\;=\;\frac{\frac{1}{nR}\sqrt{\frac{n^{2}L}{C}}}{\frac{1}{R}\sqrt{\frac{L}{C}}}\;=\;1$$

This demonstrates that the quality factor of the resonant circuit remains unchanged, while the sensor’s inductance increases *n*-fold. This suggests that, for the given coil/sensor, it is advantageous to maximize the turn diameter.

However, an important limitation arises: excessively increasing the coil turn diameter can cause mechanical instability, leading to the following:

- Irregular inclination of the turns.
- Irregular turns distribution along the coil length.
- Turn short-circuiting.

The stability of the coil’s shape and position is discussed in more detail in Chapter 5.

### 4.4. Change in Sensor Wire Diameter

If we make a coil using a wire with diameter *d*, length *l_d_*, and resistivity *ρ_d_*, where the coil length is *l_s_* and the distance between two adjacent turns is equal to *d*, the inductance *L* and resistance *R* of such a coil can be expressed as:

(11) $$\begin{array}{l} L=\frac{\mu \;z^{2}A}{l_{s}}=\frac{\mu \;{\left(\frac{l_{s}}{2d}\;\right)}^{2}A}{l_{s}}=\frac{1}{d^{2}}=\frac{\mu \;l_{s}A}{4}\\ R\;=\;\rho_{d}\;\frac{l_{d}}{a}\;=\frac{4\rho_{d}\;l_{d}}{\pi d^{2}}\;\;\;\;\mathrm{and}\;\;\;\;a\;=\;\frac{\pi d^{2}}{4} \end{array}$$

Increasing the wire diameter by a factor of *n* (to *d*_1_ = *n*·*d*) results in:

(12) $$\begin{array}{l} \mathrm{if}\;l_{s}=const.,\;z_{1}\;=\;\frac{l_{s}}{2d_{1}\left(n\right)}\;\mathrm{where}\;\;\;\;\;\;d_{1}\left(n\right)=nd\;\;\;\;\mathrm{and}\;\;\;\;a_{n}\;=\;\frac{\pi {\left(nd\right)}^{2}}{4}\\ L(n)=\frac{1}{{\left(nd\right)}^{2}}\frac{\mu \;l_{s}a_{n}}{4}=\frac{1}{n^{2}}\frac{\mu \;l_{s}a_{n}}{4d^{2}}\;\;\;\;\;\;\Rightarrow \;\;\;\;\;\frac{L(n)}{L}=\frac{1}{n^{2}}\\ R(n)\;=\frac{4\rho_{d}\;\frac{l_{d}}{n}}{\pi d^{2}}=\frac{1}{n^{3}}\frac{4\rho_{d}\;l_{d}}{\pi d^{2}}\;\;\;\;\;\Rightarrow \;\;\;\;\;\;\frac{R(n)}{R}=\frac{1}{n^{3}} \end{array}$$

Now, the change in the quality factor of the coil is given by the equation:

(13) $$Q\left(n\right)\;=\frac{n^{3}}{R}\sqrt{\frac{L}{nC^{2}}}=\frac{n^{2}}{R_{1}}\sqrt{\frac{L}{C}}\;\;\;\;\;\;\;\;\;\Rightarrow \;\;\;\;\;\;\;\;\frac{Q\left(n\right)}{Q}=n^{2}$$

Thus, we see that the quality factor of the entire circuit increases by a factor of *n*^2^. However, since the number of turns in the coil decreases by a factor of *n*, its inductance also decreases by a factor of *n*^2^. This means that the destabilizing influence of parasitic inductances increases.

The strategy of increasing wire diameter can be applied when parasitic capacitances changes (e.g., from feedthroughs) have relatively high values. By sacrificing an increase in the resonant circuit’s quality factor, it is possible to significantly increase its capacitance. Comparing *Q* from Equation (2) and *Q*(*n*) from Equation (13), we obtain:

(14) $$\;\;\frac{C\left(n\right)}{C}\;=\;n^{4}$$

As a result, the coil inductance decreases by a factor of *n*^2^, but the capacitance increases by a factor of *n*^4^.

The influence of parasitic capacitances on the measurement error can be mathematically determined based on the equations for the oscillation frequency (1) and for the sensor sensitivity (5). Using these equations, we obtain:

(15) $$\Delta l\left(C_{p}\right)=\frac{\delta f}{s}=\frac{1}{s}\left(\frac{f_{1}}{f_{2}}-1\right)=\frac{1}{s}\left(\sqrt{1+\frac{\Delta C}{C}}-1\right)\approx \frac{1}{s}\frac{\Delta C}{2C}$$

where Δ*C* represents the changes in the capacitance of the resonant circuit during measurement. In the present case, this is mainly the change in capacitance of the electrical pressure feedthroughs. The value of expression (15) can be reduced by increasing the capacitance of the resonant circuit, which results in a decrease in its quality factor and, consequently, a deterioration of oscillation stability.

In the high-pressure chamber of the GTA-10 device, the capacitance of the aforementioned feedthroughs is approximately 60 pF. During experiments in which the chamber pressure reached 400 MPa with a temperature variation of about 30 °C, this capacitance changed by a maximum of about 0.3 pF. With a resonant circuit capacitance of *C* = 1000 pF and a sensor sensitivity of 3 × 10^−5^/µm, this resulted in a measurement error of 1.7 µm. The use of a reference sensor reduced this error almost tenfold, to 0.2 µm.

However, it should be noted that increasing the diameter of the wire used for the coil (while maintaining the same coil diameter) also increases the tension force of the spring. This, in turn, has specific consequences for the mechanical system in which the sensor operates. This topic is further discussed in Chapter 5.

### 4.5. Simultaneous Change in Wire Diameter and Coil Turn Diameter

The cases described in Section 4.2 and Section 4.3 considered situations where either the coil wire diameter *d* or the coil turn diameter *D* was changed while assuming a constant sensor/coil length *l_s_*. The issue of turn stability was also mentioned (Section 3 and Section 4.2), where an excessive increase in the ratio *D*/*d* can cause the turns to lose their ability to maintain a constant distance from each other. This can lead to electrical short circuits, friction between turns, and, ultimately, instability in oscillations. To prevent this, the *D*/*d* ratio should not exceed a certain value (see Section 5), or the coil should be stretched to increase the spacing between turns. However, stretching the coil reduces the sensor’s sensitivity (see Equation (5)) or the coil may be too long compared to the measured sample length.

The following equations describe the changes in the inductance, resistance of the sensor, and quality factor of the resonant circuit, assuming a constant sensor length and a fixed *D*/*d* ratio:

(16) $$\begin{array}{l} \left.\begin{array}{l} w=\frac{D}{d}\;=\;const.\\ l_{s}=2dz\;=\;const. \end{array}\right\}\;\Rightarrow \;\;\;l_{d}=\pi Dz=\;\frac{1}{2}\pi wl_{s}=const.\\ L=\;\frac{\mu \;z^{2}A}{l_{s}}\;=\frac{\mu }{l_{s}}\;\frac{l_{s}^{2}}{4d^{2}}\;\frac{\pi D^{2}}{4}=\frac{\mu \pi }{16}\;l_{s}\;w^{2}=const.\\ R\;=\frac{\rho_{d}l_{d}\;}{a_{d}}=\frac{4\rho_{d}\;l_{d}}{\pi d^{2}}\\ Q=\frac{1}{R}\sqrt{\frac{L}{C}}\;=\;\frac{\pi d^{2}}{4\rho_{d}\;l_{d}}\;\sqrt{\frac{\frac{\mu \pi }{16}\;l_{s}\;w^{2}}{C}}\;=\;\frac{\pi d^{2}w}{16\rho_{d}\;l_{d}}\;\sqrt{\frac{\;\mu \pi l_{s}\;}{C}}\;=\;k\;d^{2}\\ \;\mathrm{and}\;\;k\;=\;\frac{\pi w}{16\rho_{d}\;l_{d}}\;\sqrt{\frac{\;\mu \pi l_{s}\;}{C}} \end{array}$$

where *l_d_* is the length of the wire used to make the sensor/coil.

From Equation (16), it follows that if *w* = *const*. and *l_s_* = *const*., then *L* = *const*. and *l_d_* = *const*. However, if *w* = *const*., the sensor resistance *R* decreases with the square of the wire diameter *d*, which necessitates a change in the coil turn diameter *D*. Consequently, the quality factor of the resonant circuit increases proportionally to *d*^2^.

Compared to the case where only the wire diameter is increased (Section 4.3), simultaneously increasing both the wire diameter and the coil turn diameter is significantly more advantageous. This is because the coil inductance remains unchanged, while the resonant circuit quality factor increases as in the previous case, that is, *n*^2^-times.

The increase in coil turn diameter is primarily limited by two factors: the first—the limited space available in the pressure chamber (see Section 1, Figure 3), and the second—the increase in force acting on the sensor’s mounting hooks, which grows as *d*^4^ to the increase in wire diameter.

The issue of forces acting on the mounting hooks is discussed in the next section.

## 5. Tensioning Force of the Sensor

The tensioning force *F* of our coil/spring is directly proportional to its elongation Δ*l_s_*, according to the formula [50]:

(17) $$F\;=\;-k\;\cdot \;\Delta l_{s}$$

The constant *k* for a spring is given by [50]:

(18) $$k\;=\;\frac{G_{s}\;d^{4}}{8\;D^{3}\;z}$$

where *G_s_* is the shear modulus (Kirchhoff modulus) of the spring material, and the remaining symbols are as previously defined. When the wire diameter is increased *n*-fold (*d*_1_ = *n*·*d*), then:

(19) $$\frac{k\left(d_{1}\right)}{k\left(d\right)}\;=\;\frac{{d_{1}}^{4}}{d^{4}}\;=\;{\frac{\left(n\cdot d\right)}{d^{4}}}^{4}\;=\;n^{4}$$

which means that increasing the wire diameter *n*-fold results in a *n*^4^-fold increase in the spring constant *k* and, consequently, the force exerted on the sensor mounting system.

Now, let us consider a sensor/spring with an initial length (i.e., unstretched) of *l*_0_. The tension force of this sensor is given by the equation:

(20) $$F\;=\;-\frac{G_{s}\;d^{4}}{8\;D^{3}\;z}\;\cdot \;\Delta l_{s}$$

This sensor meets the requirement of maximizing its inductance while maintaining sufficient spacing between turns when the distance between them after stretching equals the wire diameter. This implies that Δ*l_s_* = *l*_0_. Since *w* = *D*/*d* (Equation (16)), we obtain:

(21) $$F\;=\;-\frac{G_{s}\;d^{4}}{8\;D^{3}\;z}\;l_{0}\;=\;-\frac{G_{s}\;d^{4}}{8{\left(wd\right)}^{3}\frac{l_{0}}{d}}\;l_{0}\;=\;-\;\frac{G_{s}\;d^{2}}{8\;w^{3}}$$

**Figure 5:**
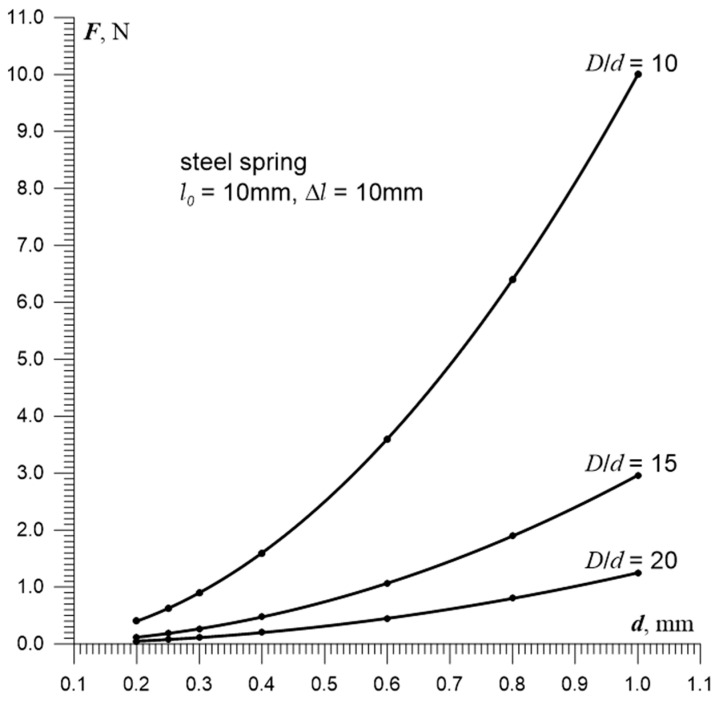
Tension force of the sensor/spring vs. wire diameter.

Figure 5 presents the relationship between the tension force of a steel spring and the wire diameter, derived from Equation (21). Calculations were performed assuming: *l*_0_ = 10 mm, Δ*l_s_* = *l*_0_, 0.20 mm ≤ *d* ≤ 1.0 mm, *w* = {10, 15, 20}, and *G_s_* = 85.0 GPa. These dependencies show that with a fivefold increase in wire diameter, the maximum force *F* is:

- For so-called “hard” springs *F* = 10.6 N (*w* = *D*/*d* = 10).
- For “soft” springs *F* = 1.3 N (*w* = 20).

At this point, it is necessary to determine what value of sensor tension force is still acceptable and what should be considered excessive. This is not an easy issue to resolve. If we focus solely on a qualitative description, an excessive force is one that:

- Causes the distance between the mounting hooks securing the spring to the sample to become unstable (due to material creep in the hooks’ material).
- Causes material creep in the sensor itself, leading to changes in sensor geometry and destabilization of the electrical parameters of the system.

Attempting to quantify the issue, the authors of the article found that during rock compressibility tests (i.e., measuring changes in the sample’s volume under hydrostatic pressure up to 400 MPa), the resolution for measuring the sample’s linear deformation was better than 10^−5^.

In the authors laboratory measurements, a sensor with *d* = 0.2 mm and *w* ≈ 12 was used, mounted to the hooks using soldering and tensioned with a force of approximately 0.25 N, which is considered acceptable even when the hooks are glued to the sample.

From Figure 5, it can be inferred that increasing the wire diameter *d* from 0.20 mm to 0.36 mm reduces its resistance by a factor of 3.6, which theoretically—according to Equation (3)—allows a tenfold increase in the capacitance of the resonant circuit. As a result, the stability of oscillations under varying pressure and temperature conditions would be significantly improved.

## 6. The Problem of Coil/Sensor Turns Stability

**Figure 6:**
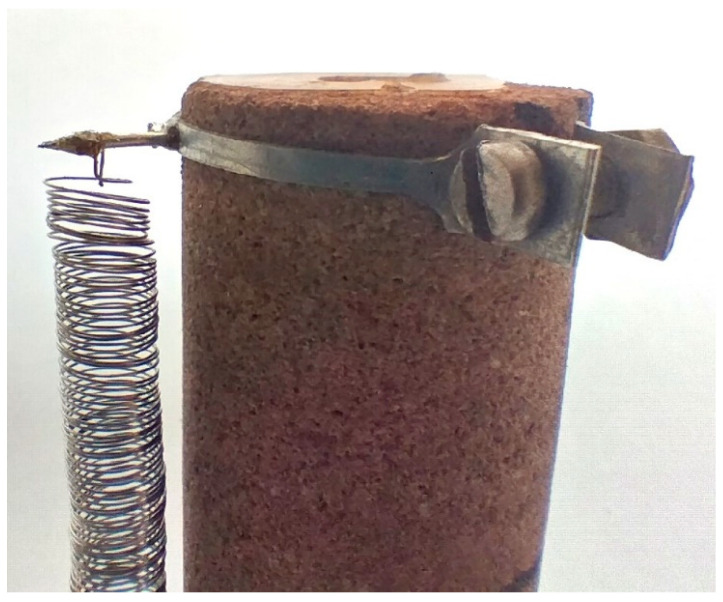
Example of loss of parallelism of the coil turns near the fixing point; the coil is fixed to the specimen with a screw-on clamp.

It is desirable that the sensor is as short as possible which provides high sensitivity, and its turns remain as close together as possible during measurement which ensures high inductance. If the sensor length is fixed, its inductance can also be increased by enlarging the coil diameter *D*. However, excessively increasing the coil diameter while maintaining a constant wire diameter *d* leads to a situation where the coil turns are no longer parallel to each other (they start to “warp”) or even come into contact with each other (as it is shown on Figure 6). This results in a loss of oscillation stability.

It seems impossible to define a strict limit for the acceptable ratio of coil diameter *D* to the wire diameter *d*. A website dedicated to designing tension springs [51] refers to the coefficient *w* = *D*/*d* as the “coil ratio” and estimates its maximum value as:

(22) 14 ≤ w ≤ 20

If *w* ≤ 10 (the recommended value for springs in mechanical applications), the authors’ experience suggests that the problem of turns stability does not arise. Much depends on the precision of the spring’s manufacturing, i.e., ensuring constant wire tension while coil winding, maintaining a uniform winding speed, and minimizing wire deformation before winding. One could assume that if all coil turns conditions and wire parameters were ideal, there would be no theoretical upper limit for the coefficient *w*.

One of the authors of this paper demonstrated in doctoral research that the coil diameter should not exceed 15 to 20 times the thickness of the wire from which it is made. This means that for a wire with a diameter of 0.2 mm, which is currently used for sensors in the laboratory, the maximum sensor/spring coil diameter should be within: 3.0 mm ≤ *d* ≤ 4.0 mm (provided that the spring is carefully manufactured).

When designing the spring, it is important to remember that if it is to function as a deformation sensor, its tension force must not be “too high” in the sense defined by conditions (a) and (b) in Section 5. In particular, for the aforementioned wire with *d* = 0.2 mm and a so-called “soft spring” (15 ≤ *D*/*d* ≤ 20), this force is approximately 0.1 N (see Figure 5).

## 7. Influence of Temperature Variations on the Operating Frequency of the Transducer

As a consequence of temperature changes in the measurement system, both the length of the wire forming the sensor/coil and its electrical resistance will vary. The influence of these variations on the operating frequency of the transducer has been thoroughly addressed by the authors in previous works [12,52]. For the purposes of this paper, the solution to this problem is presented in a concise form so as not to unduly extend the length of the article.

If the sensor is mounted on an object with zero thermal expansion, the change in its diameter *D* will be proportional to the elongation of the wire. Assuming that the coefficient of thermal expansion of the sensor wire is *α_l_*, and that the remaining parameters of the resonant circuit remain unchanged (see Equation (1)), we obtain:

(23) $$\begin{array}{l} \frac{\Delta f\left(\alpha_{l}\right)}{f_{0}}\;=\;\frac{f_{T}}{f_{0}}\;-\;1\;=\;{\left(\frac{A_{0}}{A_{T}}\right)}^{0.5}\;-\;1\;=\;\frac{D_{0}}{D_{T}}\;-\;1\;=\;\frac{D_{0}}{D_{0}\;\left(1\;+\;\alpha_{l}\;\Delta T\right)}\;-\;1\;=\\ \;=\;\frac{1}{1\;+\;\alpha_{l}\;\Delta T}\;-\;1\;=\;\frac{-\alpha_{l}\;\Delta T}{1\;+\;\alpha_{l}\;\Delta T}\;\approx \;-\alpha_{l}\;\Delta T \end{array}$$

In Equation (23), the subscripts 0 and *T* denote, respectively, the initial value of the variable and its value after a temperature change in Δ*T*. Thus, the relative variations in the operating frequency of the transducer will be approximately equal to the product of the system temperature change Δ*T* and the thermal expansion coefficient of the coil wire *α_l_*, which for most metals is on the order of 10^−5^/°C.

The influence of temperature on strain measurements performed with the presented sensor/coil can be minimized in five ways:

1. Limiting temperature variations during transducer operation—although this is not always feasible.
2. Introducing a mathematical correction to the measured data—this requires knowledge of the thermal characteristics of the sensor and sufficiently accurate measurements of temperature changes.
3. Compensating the temperature effect on the sensor by incorporating into the measurement circuit a capacitor with a thermal capacitance coefficient chosen to counterbalance the thermal influence on the sensor/coil. This approach is feasible only when temperature changes occur slowly, and both the coil and the capacitor are exposed to the same thermal environment. For rapid temperature fluctuations, the different thermal inertias of the coil and the resonant-circuit capacitor must be taken into account.
4. Designing the resonant circuit such that, due to the oscillation frequency, the thermal variation in the sensor inductance is compensated by the thermal variation in its resistance.
5. Application of a reference sensor—comparative measurement method.

As the capacitors of the resonant circuit are located in an environment different from that of the sensor/coil, thermal compensation of the transducer can be implemented through appropriate resonant circuit design (see above, point 4), combined with the application of the comparative measurement method (point 5) and the incorporation of mathematical correction of the measured results (point 2). Assuming that *α_R_* is the temperature coefficient of resistance of the sensor, *R* its resistance, and Δ*T* the temperature variation in the measurement system, we obtain:

(24) $$\frac{f_{T}}{f_{0}}\;=\;1\;+\;\frac{l_{s}}{\mu \;z^{2}\;A}\;2\;\alpha_{R}\;R\;\left(\frac{C_{L}^{2}}{C_{1,2}}\;+\;C_{L}\;+\;\frac{C_{1,2}}{4}\right)\;\left(R\;\alpha_{R}\;\Delta T^{2}\;+\;R\Delta T\right)\;-\;\alpha_{l}\;\Delta T$$

(other notations are as in Figure 3 and Equation (1)). This is a parabolic equation of the form y = a·(b·Δ*T*^2^ + c·Δ*T*), where the variable is the temperature change Δ*T*.

**Figure 7:**
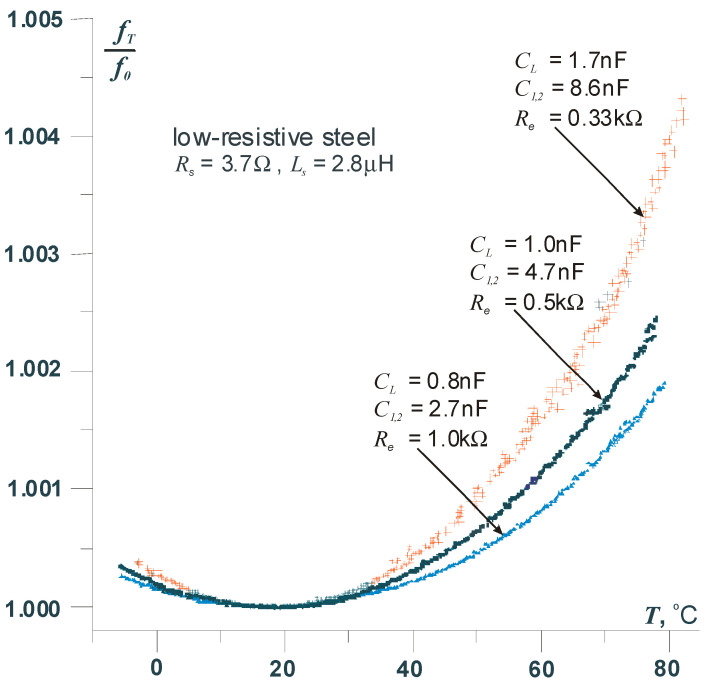
Thermal characteristics of the sensor (measurement curves); the results obtained for low-resistance carbon steel sensors and different values of resonant circuit capacitors.

By selecting appropriate capacitor values, it is possible to achieve compensation of the temperature effect on the frequency within the temperature range in which the measurement is performed. The key to achieving thermal compensation lies in modifying the resonant circuit by adding a capacitor *C_L_*, which shunts the capacitive divider *C*_1_, *C*_2_ (see Figure 1). Detailed information on this compensation method is provided in [11,12]. It should be emphasized that the influence of temperature changes on the sensor outside the compensation point will increase, which is a direct consequence of the parabolic dependence of the frequency on the sensor temperature. To minimize this effect, for example, in measurements requiring very high precision, the use of a reference sensor is necessary. To illustrate the above issue, Figure 7 shows the thermal characteristic of the transducer operating in the modified resonant circuit.

## 8. Summary

If the sensor measures sample deformations inside a high-pressure cell—operating, in this case, at hydrostatic pressures of up to 400 MPa—it is connected to an external LC oscillator via electrical feedthroughs in the cell’s plug. In such a setup, the primary factor destabilizing the oscillator frequency is the influence parasitic capacitance of these feedthroughs on the resonant circuit. The value of this capacitance depends on changes in hydrostatic pressure and the accompanying temperature variations inside the cell. To minimize the destabilizing effect of these parasitic capacitances, it should be to achieve the highest possible values of the capacitors in the resonant circuit while maintaining a sufficiently high *Q*-factor of the circuit. This quality factor determines the required amplitude as well as the stability of the oscillations.

Increasing the capacitance of the resonant circuit without excessively reducing its quality factor can be achieved by increasing the inductance of the sensor while minimizing any rise in its resistance. The easiest way to increase inductance is by minimizing the spacing between the sensor’s turns, but without causing short circuits. Increasing the number of sensor’s turns to raise inductance is an unfavourable strategy because it ultimately lowers the quality factor of the circuit and reduces the sensitivity of the sensor.

Another issue is the necessity of miniaturizing the sensor due to the limited volume of the pressure chamber, particularly the small distance between the chamber wall and the compressed sample (see Section 1, Figure 2).

From the content of the article, it follows that in the design process, one should aim to create a short sensor, which—as shown by Formula (5)—will be a highly sensitive measuring instrument. It is also desirable for the sensor to have the highest possible turns density, with a coil diameter large enough to be acceptable in terms of sensor miniaturization and to ensure a stable position of the coil turns (i.e., a constant mutual inclination).

The stability of the sensor’s turns positions depends on the *D*/*d* ratio, where *D* is the coil diameter and *d* is the wire diameter. In springs designed for purely mechanical applications, this ratio is generally not greater than 10, ensuring stable turns positioning in terms of spacing between them.

To maximize inductance in the sensor, the optimal D/d ratio should be within the range: 15 < *D*/*d* < 20. If *D*/*d* > 20, the turns may become unstable, leading to short circuits and friction between them, and if *D*/*d* < 15, the inductance of the sensor is unnecessarily reduced. A sensor within the optimal range 15 < *D*/*d* < 20 ensures stable turns positioning and maximized inductance while maintaining relatively low resistance—beneficial for the quality factor of the resonant circuit. Additionally, such a sensor, when considered as a tension spring, exerts minimal force on the mounting hooks that attach it to the test sample. Achieving the maximum *D*/*d* value of approximately 20 is possible only if the spring is wound with high precision.

The best strategy for designing the transducer seems to be an initial estimation of the expected variations parasitic inductances and capacitances that may occur during measurements. Then, based on the values of these estimates, a suitable sensor geometry should be selected. This geometry, often the result of a compromise between sensor miniaturization and its inductance, should ensure that the sensor’s inductance and the lumped capacitance of the resonant circuit are sufficiently large relative to the variations in parasitic inductance and capacitance, so that—with an appropriate quality factor—the displacement can be measured with the required accuracy.

The discussions and mathematical formulas presented in this article should significantly aid in achieving this goal.

## 9. Conclusions

This paper presents the fundamental principles of designing a displacement-to-frequency transducer intended for high-resolution, wide-range displacement measurements of rock samples under pressures on the order of several hundred MPa. The design process considered the following factors:

✓ The dependence of the resonant circuit quality factor and sensor/coil sensitivity on the geometric parameters of the coil, including its length, diameter, number of turns, and the wire diameter.
✓ The influence of parasitic inductances and capacitances arising in the resonant circuit of the transducer.
✓ The relationship between the tensile force of the sensor and its geometric parameters, as well as their impact on the mechanical stability of the sensor turns.

Further development of the transducer may proceed along several directions:

➢ Designing sensor variants suitable for displacement measurements under conditions other than a high-pressure chamber, with particular emphasis on potential field applications outside the laboratory.
➢ Identifying materials with improved mechanical and electrical properties to enable the construction of sensor/coil with enhanced metrological performance.
➢ Extending the method to measurements of physical quantities other than displacement, for example, a spring balance may simultaneously serve as the inductive element of a weight-to-frequency transducer.

The transducer demonstrates high metrological performance, while remaining cost-effective and straightforward to manufacture. This work is intended to provide practical design guidelines for researchers and engineers interested in constructing such a transducer.

## 10. Patents

The following patents were developed during the work on the inductive displacement sensor:

– No. 175088. A device for measurement of linear displacement of solid material samples (by J. Nurkowski).
– No. P.410608 Method and device for measuring changes in the length of an object using an inductive sensor (by J. Nurkowski).

## Data Availability

Dataset available on request from the authors.
